# Supplementary material for: Regulatory analysis of root architectural and anatomical adaptation to nitrate and ammonium in Brachypodium distachyon
Source: Front Plant Sci. 2025 Dec 12;16:1708928. doi: 10.3389/fpls.2025.1708928 (PMC12741136; doi:10.3389/fpls.2025.1708928)
Supplement: Supplementary file 5 [file Supplementaryfile1.docx]

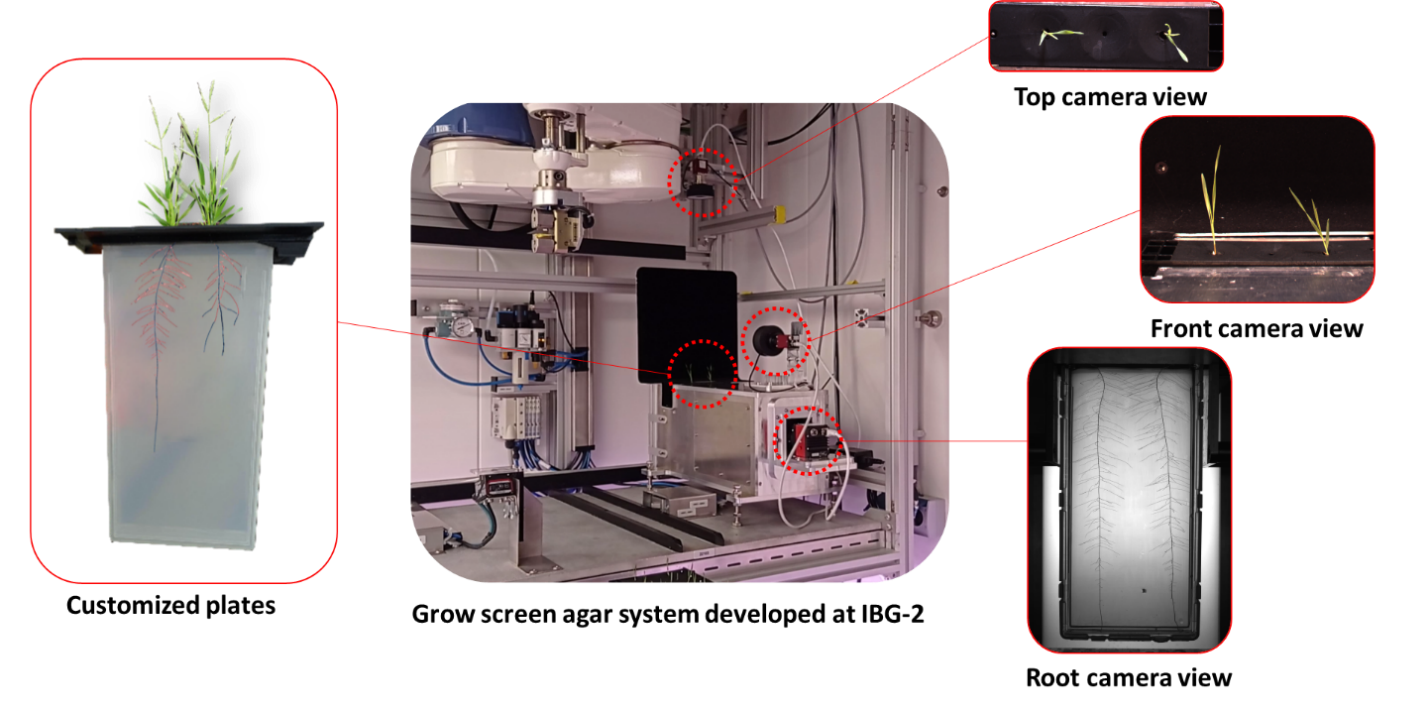
**Supplementary figure 1**


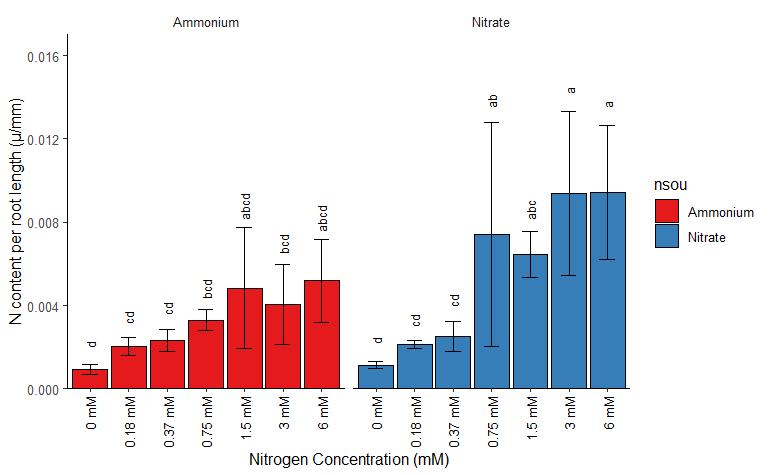
**Supplementary figure 2**


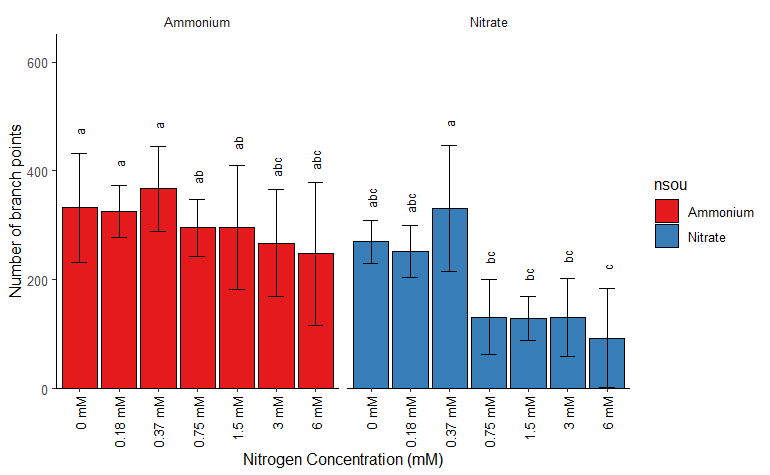

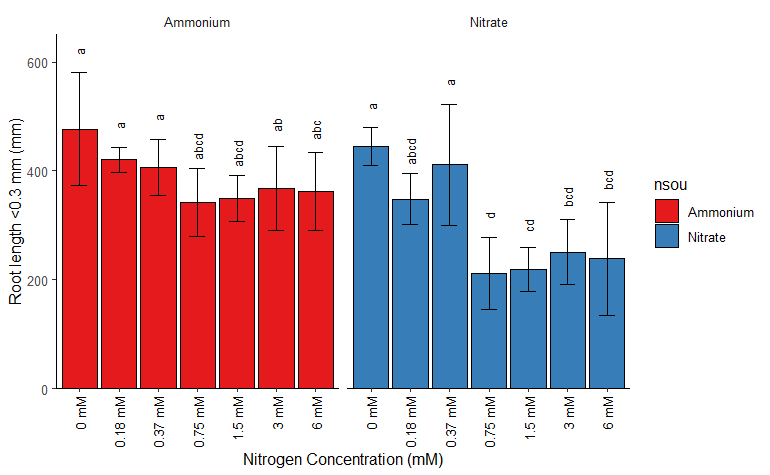

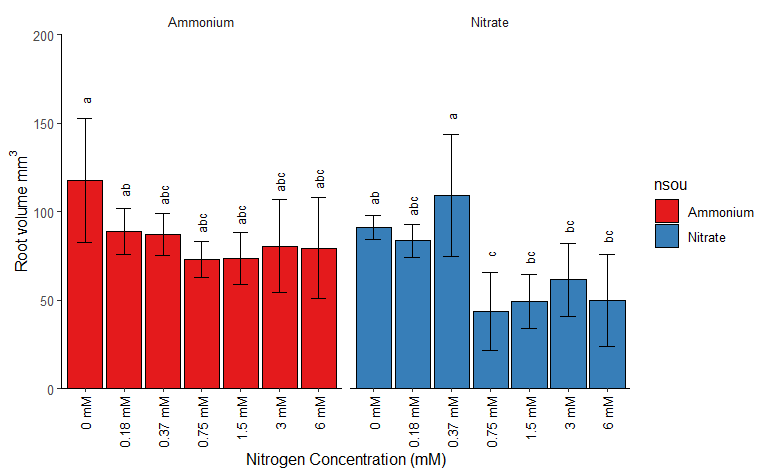

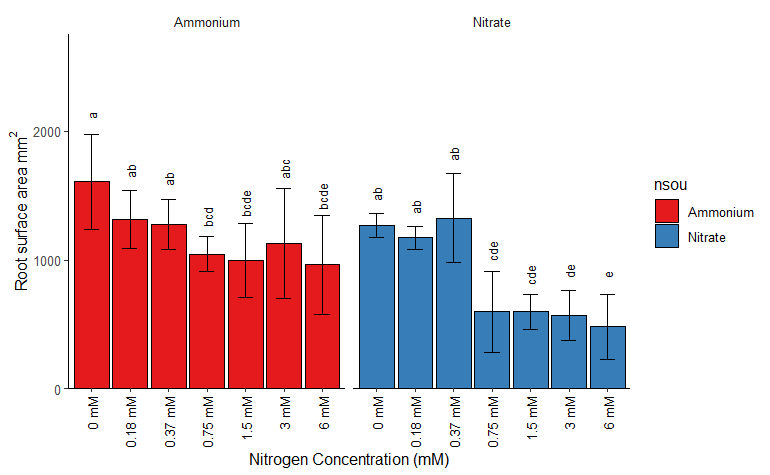
**Supplementary figure 3**

**Supplementary figure 4**


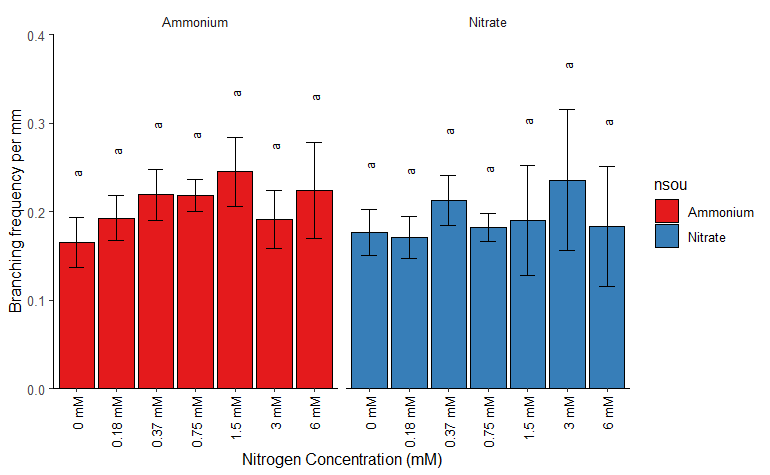


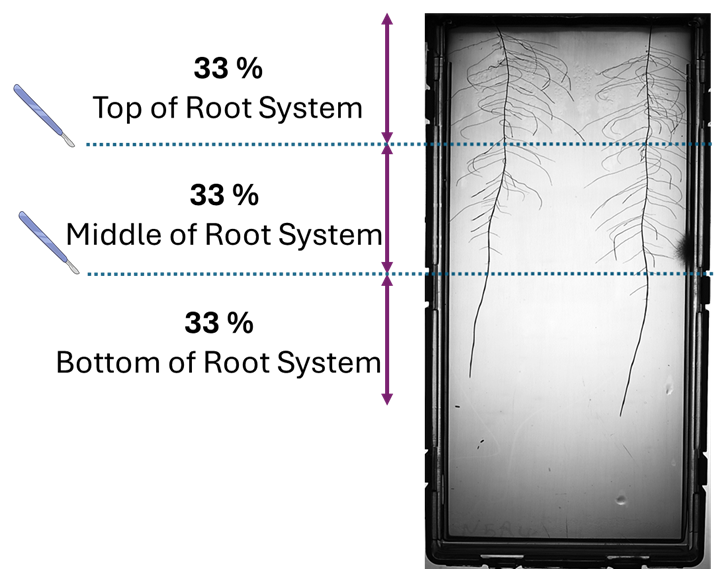
**Supplementary figure 5**

**Supplementary figure 6**


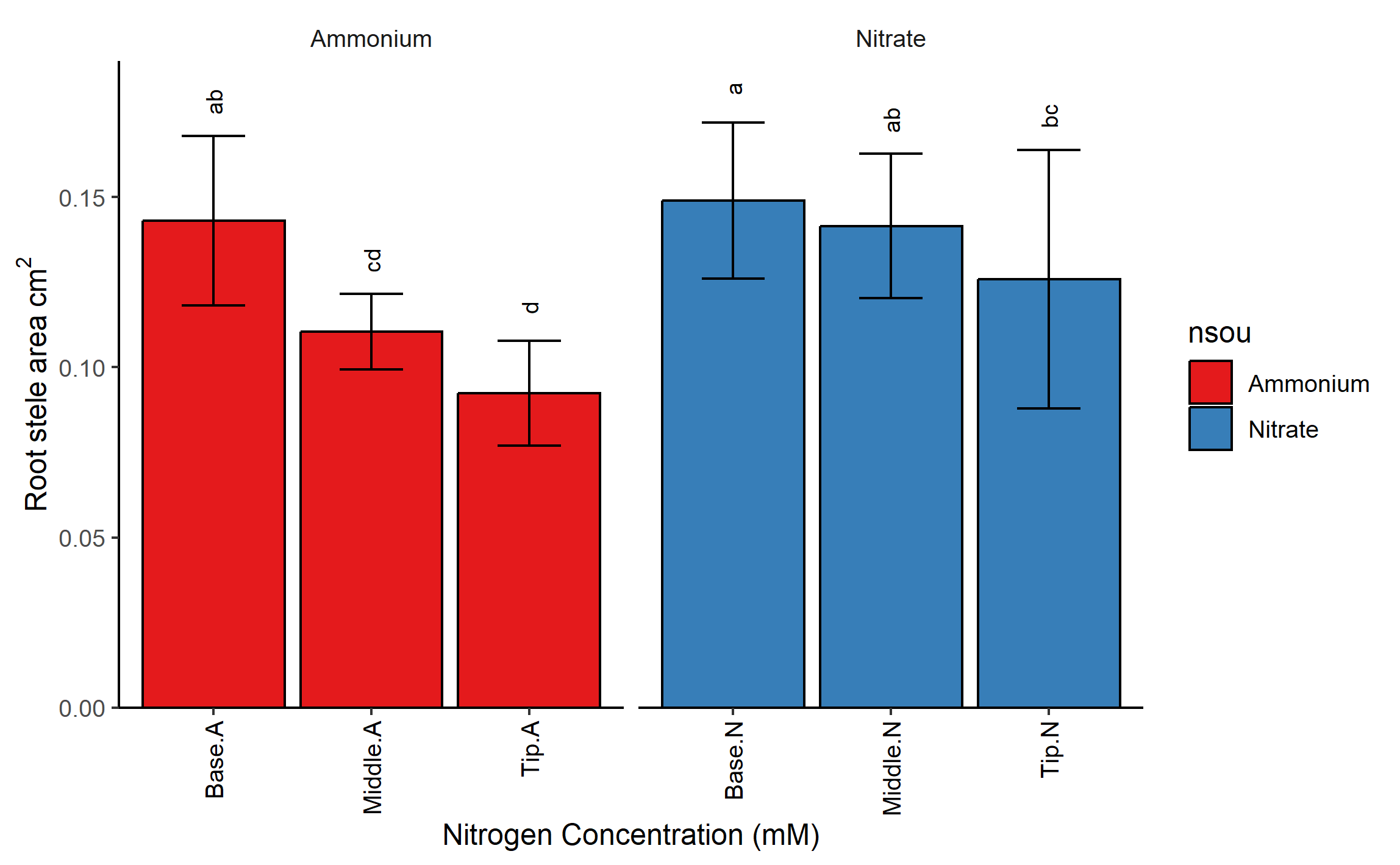

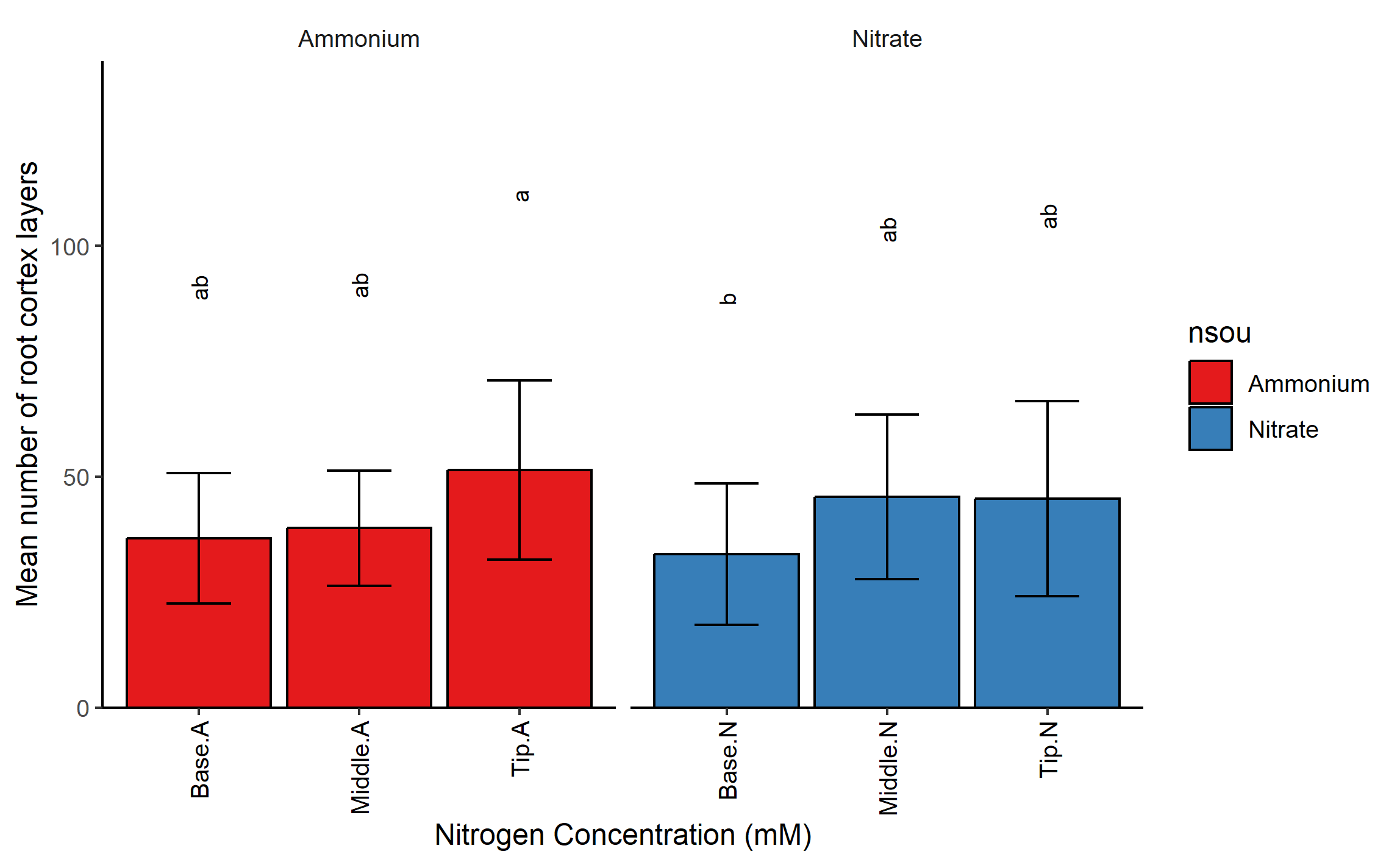


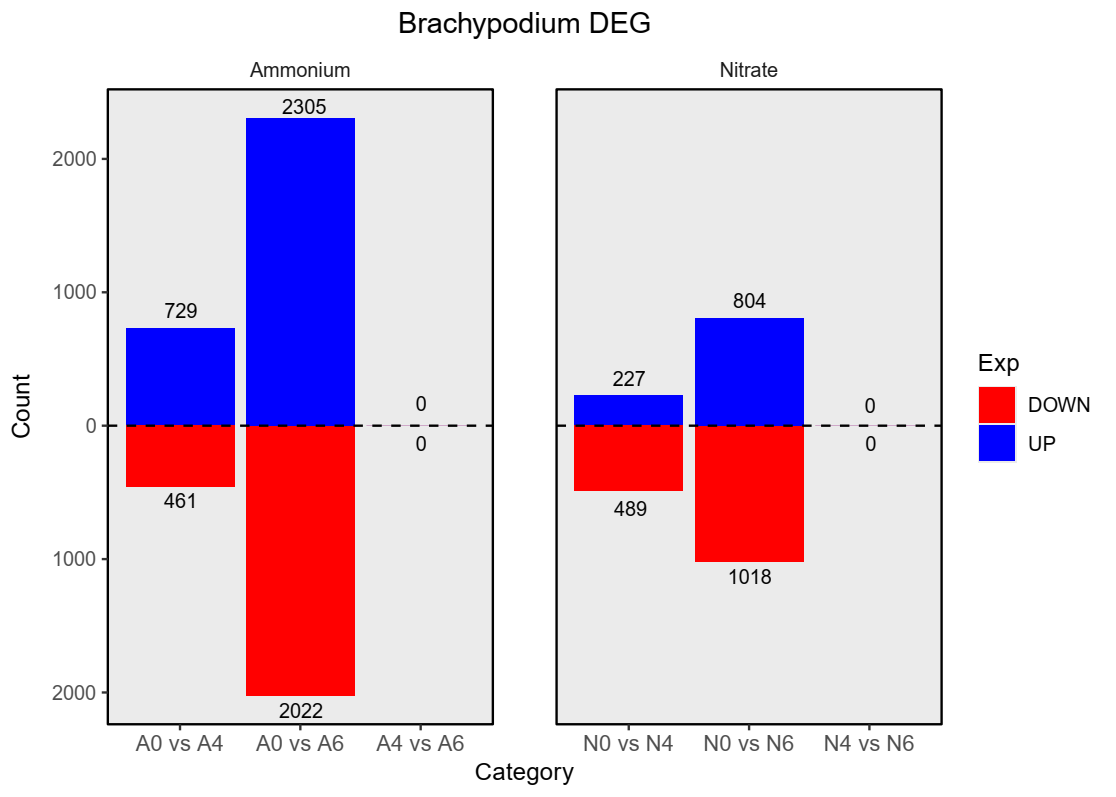
**Supplementary figure 7**


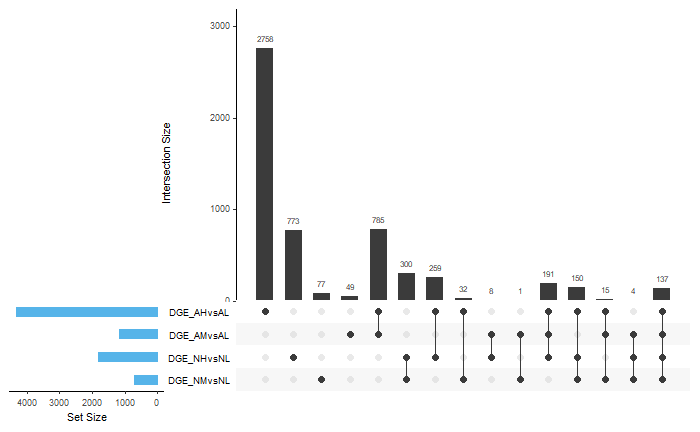


**Supplementary figure 8**

**
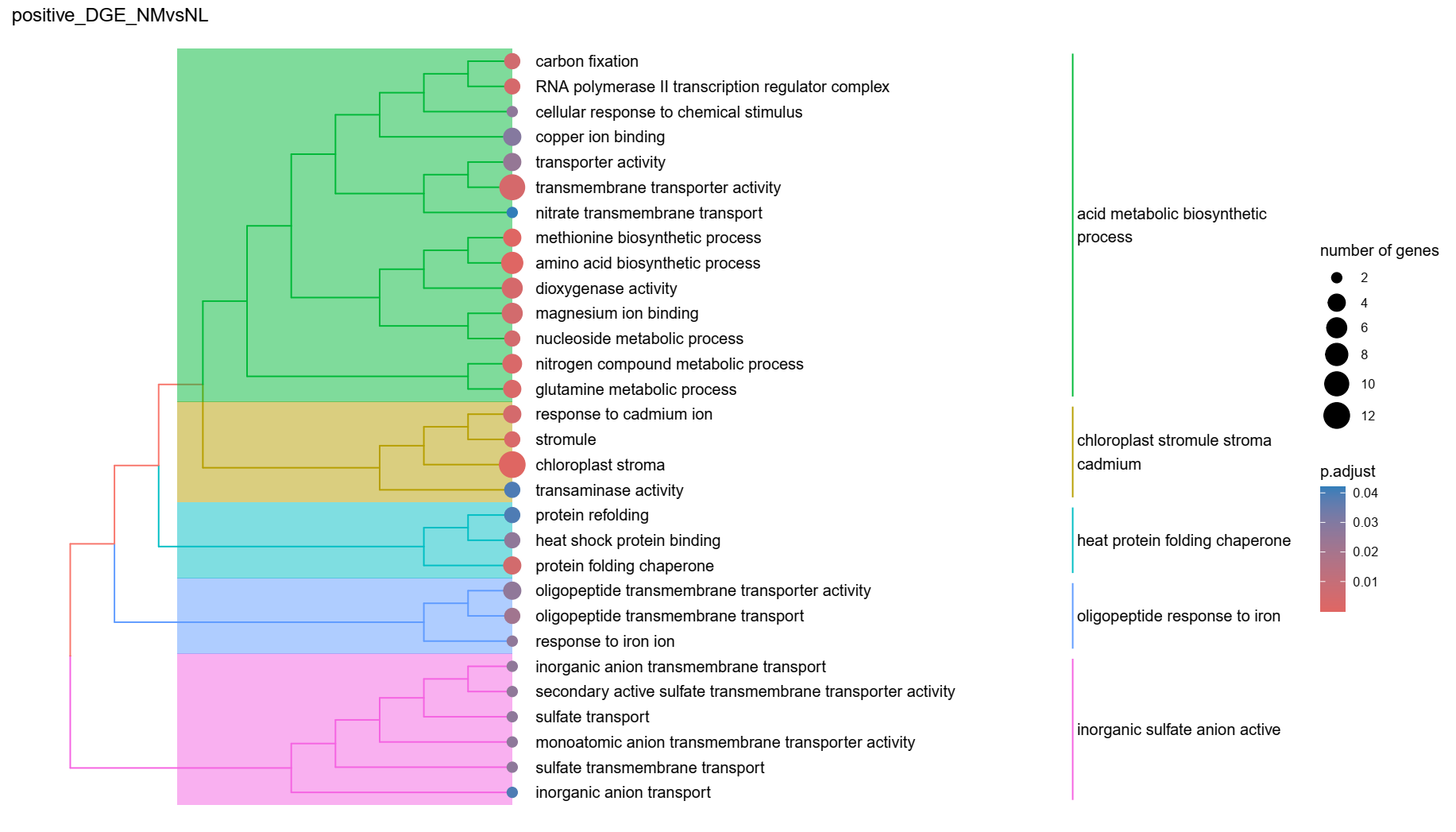
GO enrichment of up regulated genes in moderate vs low nitrate**

**
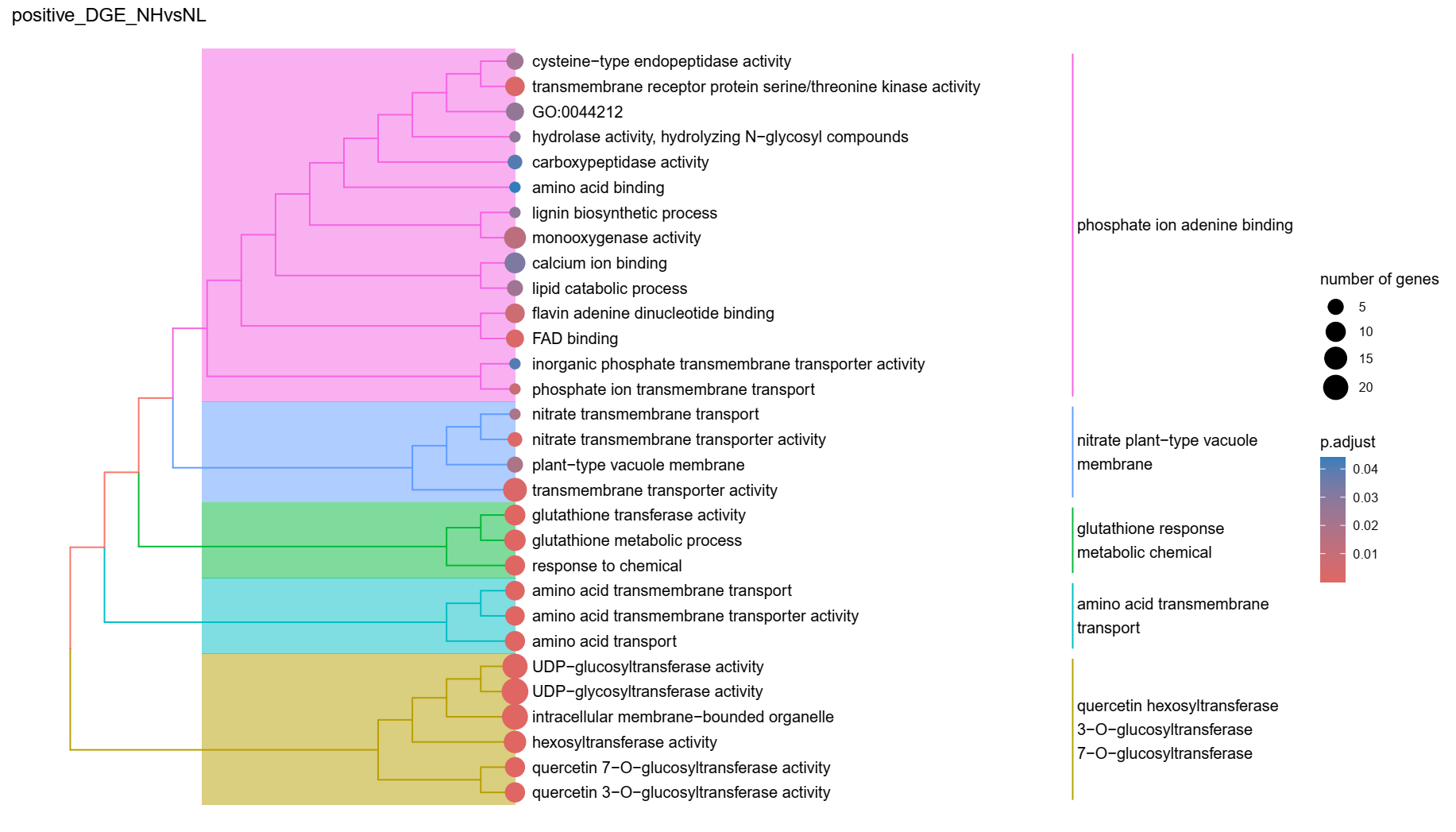
GO enrichment of up regulated genes in high vs low nitrate**


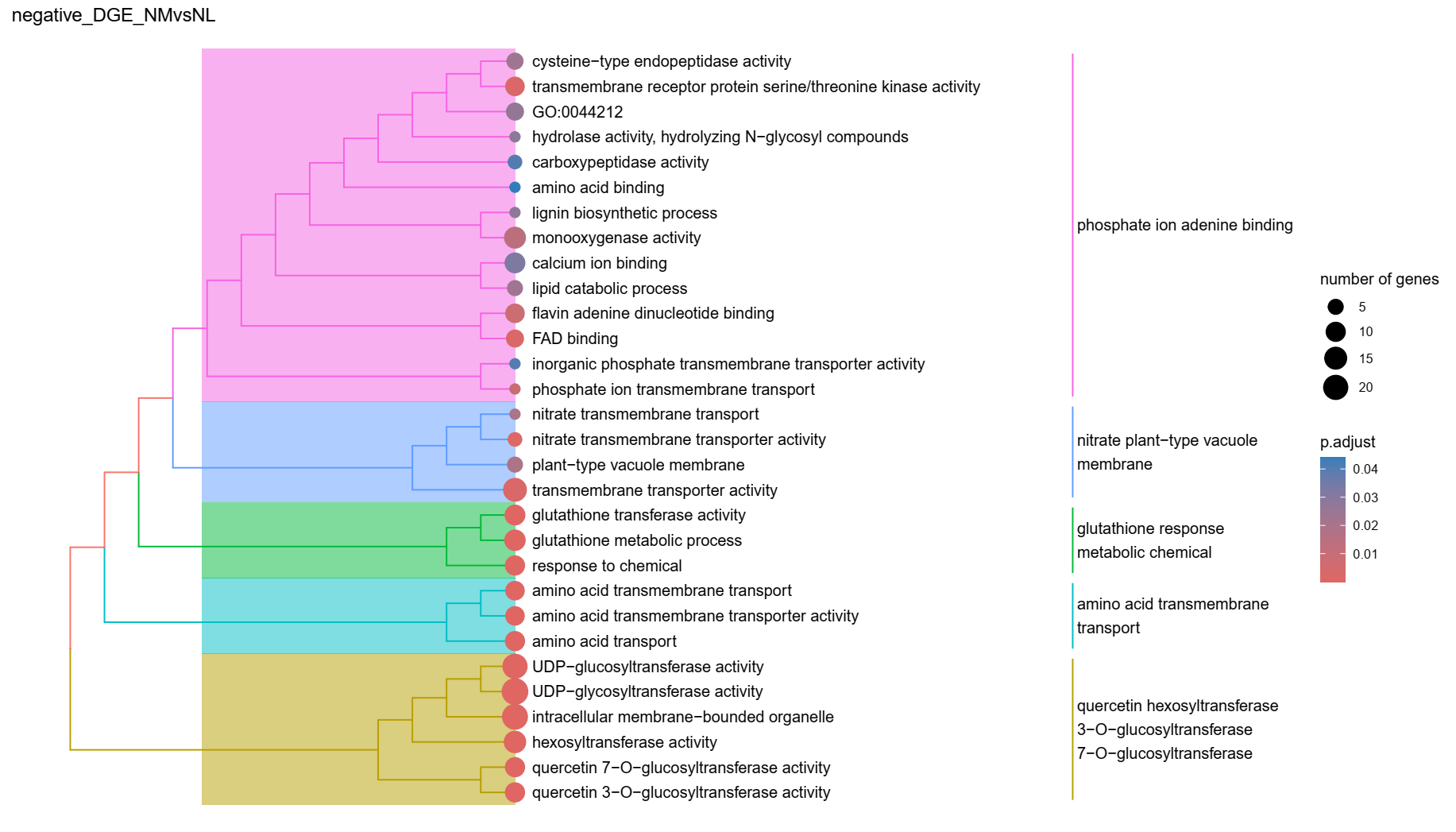
**GO enrichment of down regulated genes in moderate vs low nitrate**


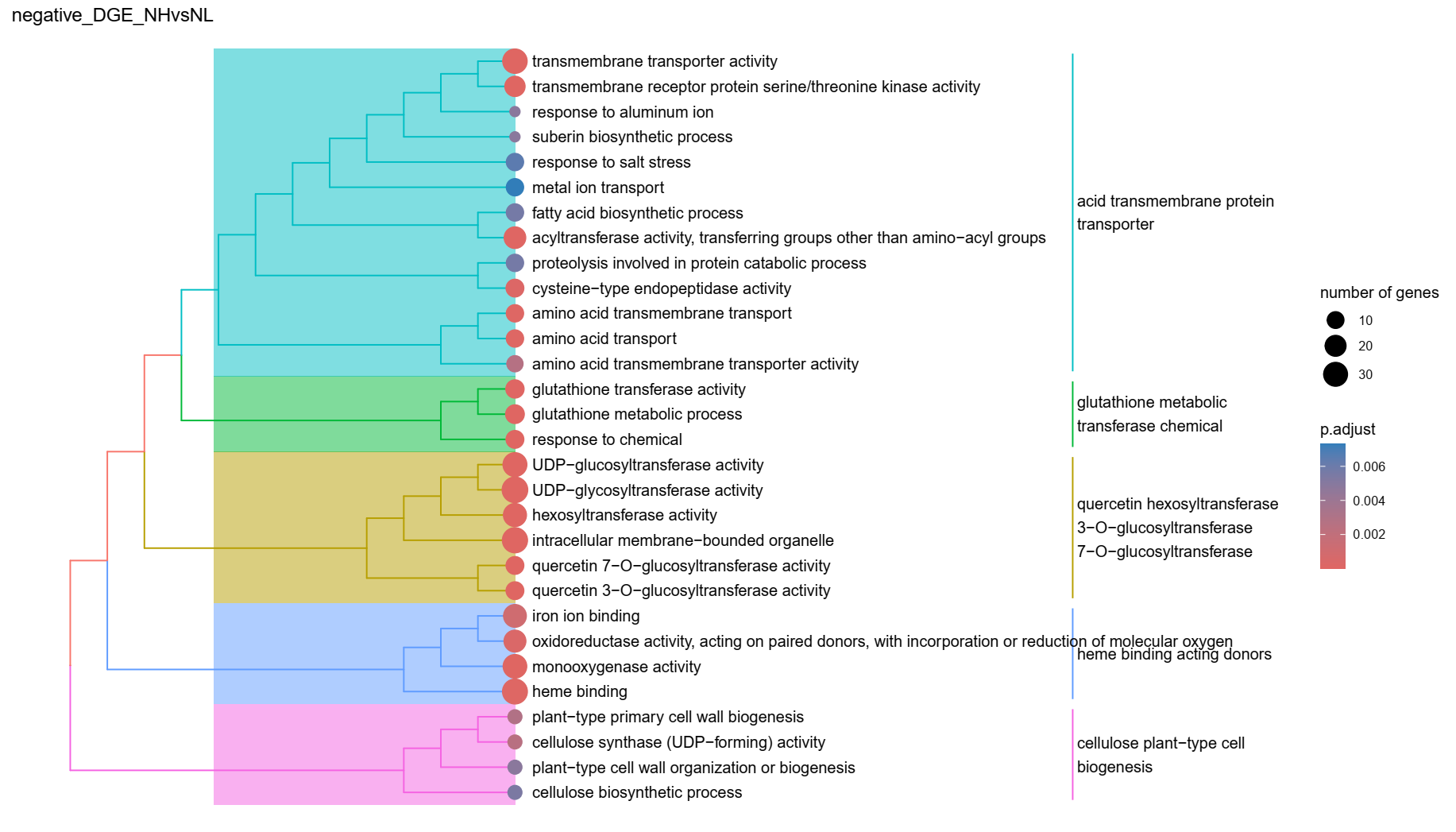
**GO enrichment of down regulated genes in high vs low nitrate**


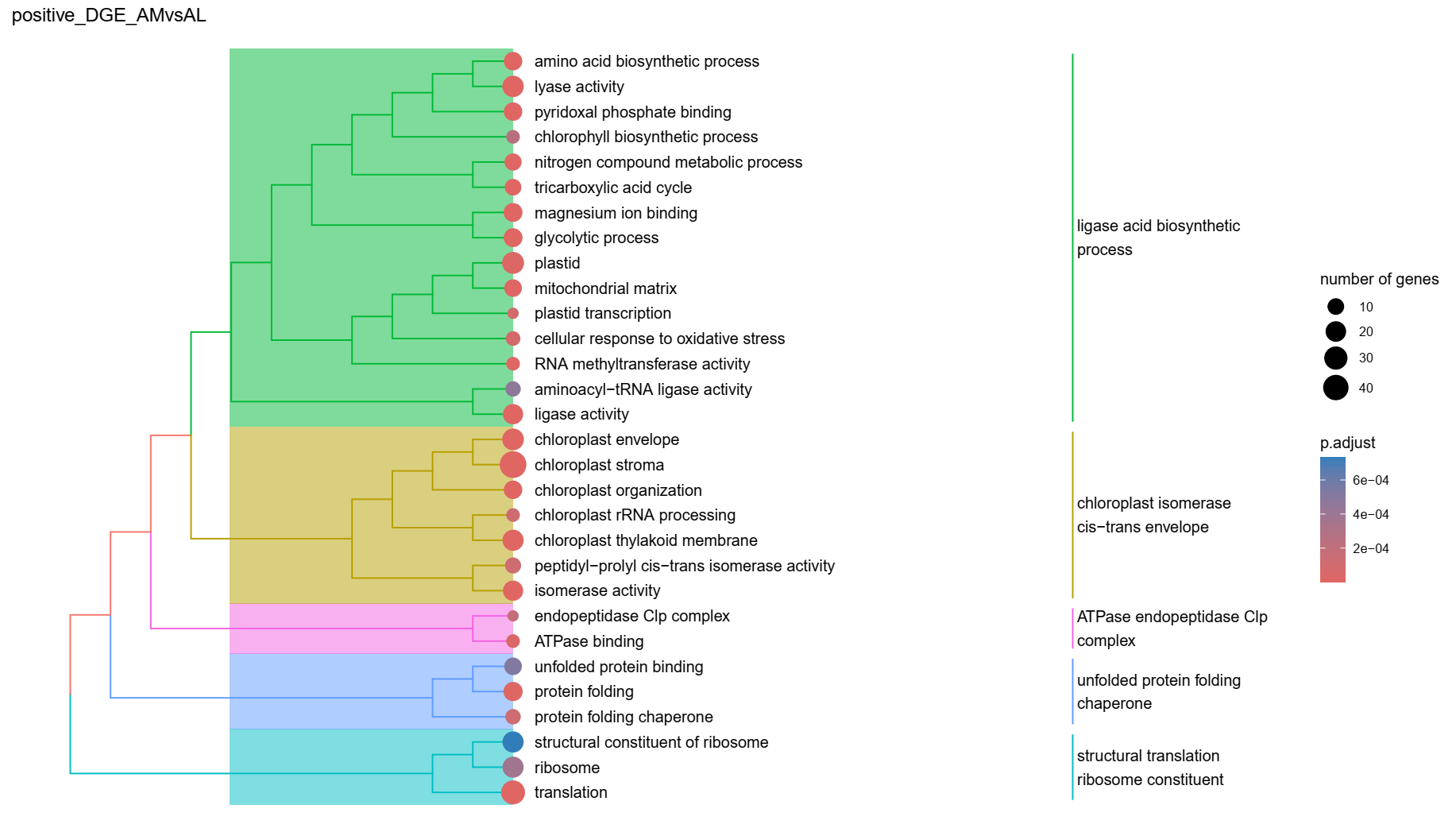
**GO enrichment of up regulated genes in moderate vs low ammonium**


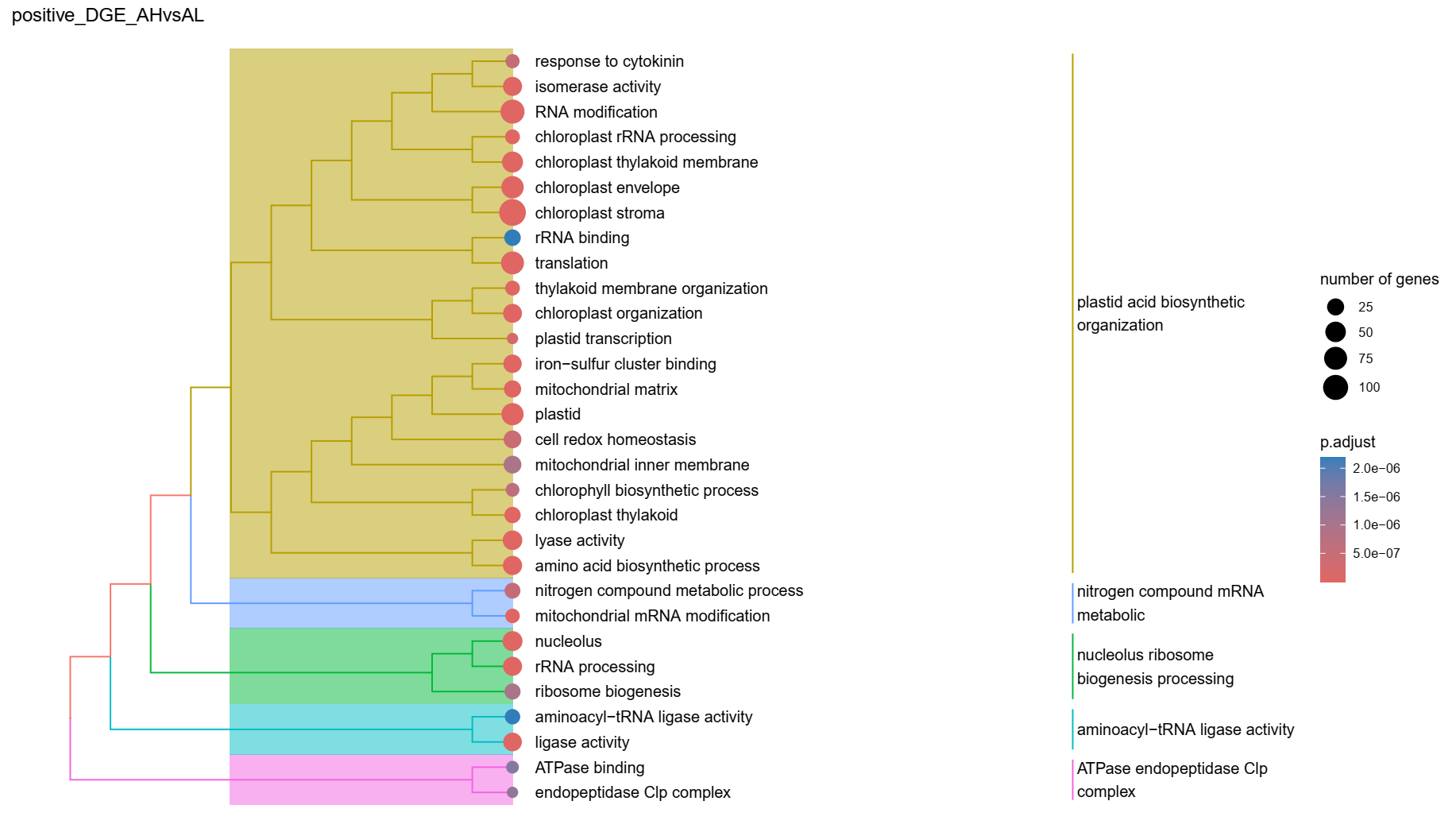
**GO enrichment of up regulated genes in high vs low ammonium**


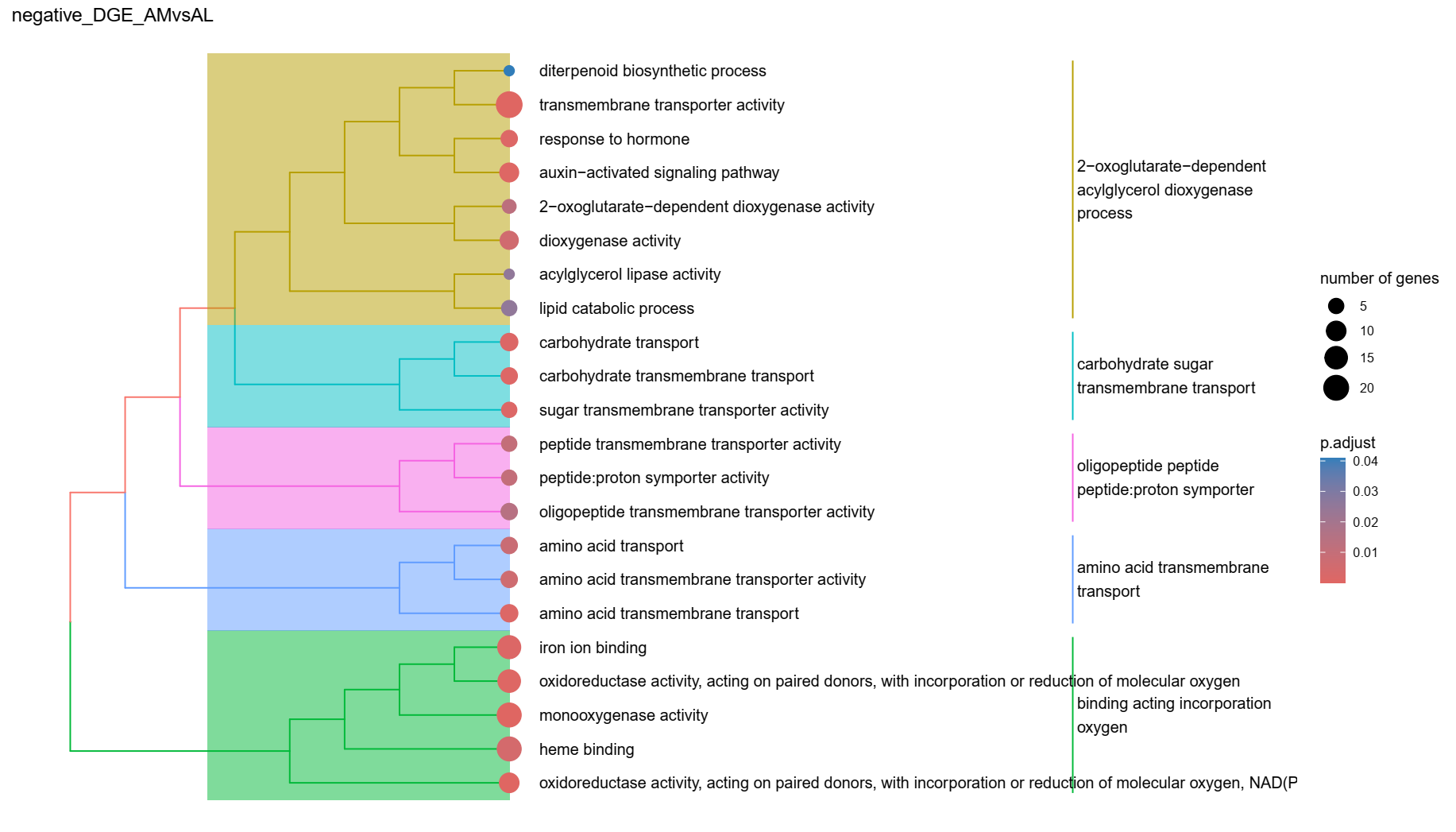
**GO enrichment of down regulated genes in moderate vs low ammonium**


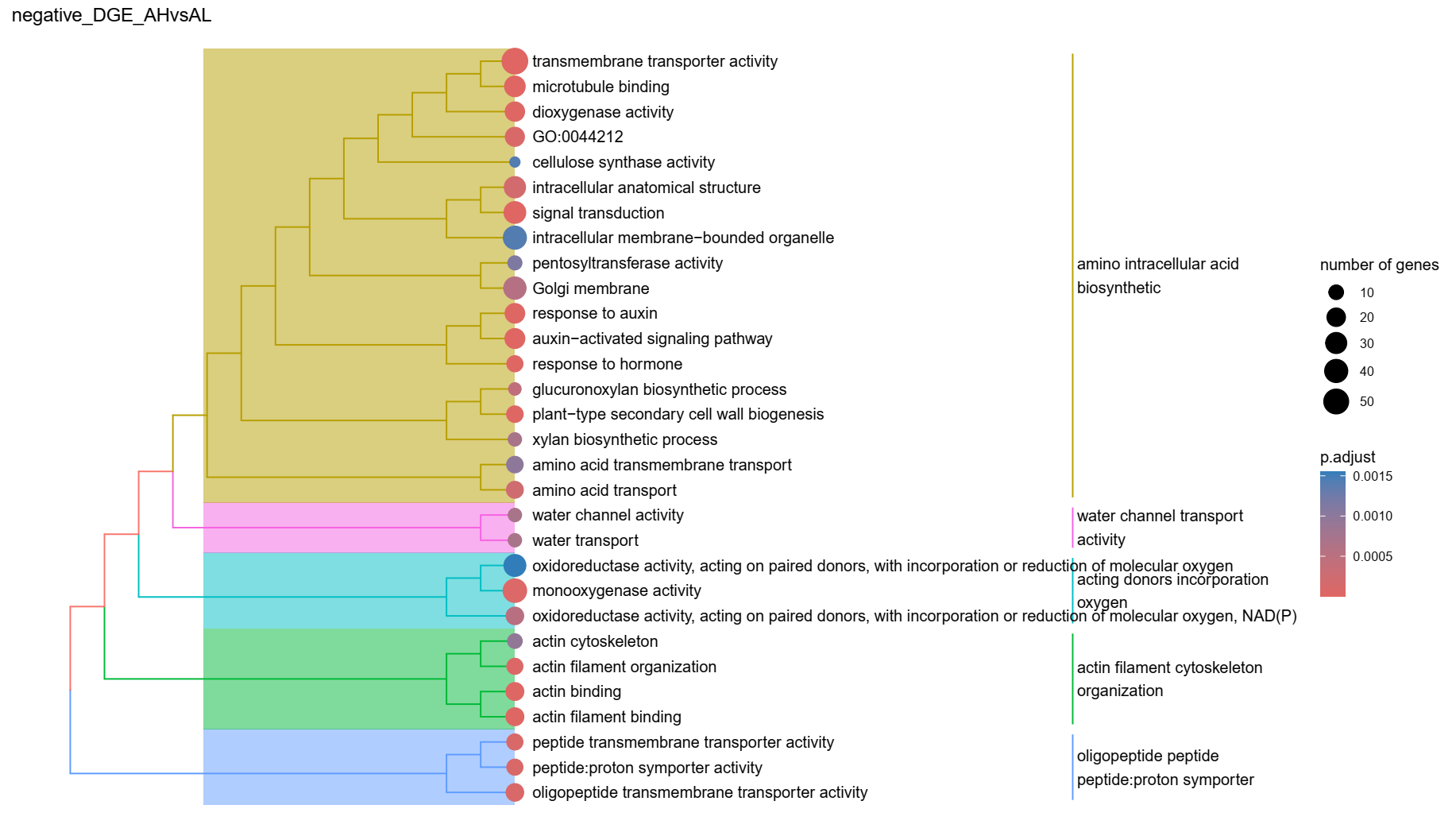
**GO enrichment of down regulated genes in high vs low ammonium**

**Supplementary Table 1**

| **Name** | **Gene ID** |
| --- | --- |
| NRT1.1 | BRADI_3g33040v3 |
| NRT1.2 | BRADI_1g37330v3 |
| NRT1.3 | BRADI_3g47010v3 |
| NRT1.4 | BRADI_2g41060v3 |
| NRT1.5 | BRADI_3g53380v3 |
| NRT2.1 | BRADI_3g01270v3 |
| NRT2.2 | BRADI_3g01250v3 |
| NRT2.3 | BRADI_3g01277v3 |
| NRT2.4 | BRADI_3g01290v3 |
| NRT2.5 | BRADI_2g47640v3 |
| NRT2.6 | BRADI_2g26210v3 |
| NRT2.7 | BRADI_2g40740v3 |
| NRT3.1 | BRADI_3g47710v3 |
| NRT3.2 | BRADI_3g47720v3 |

**Supplementary figure 9**

**
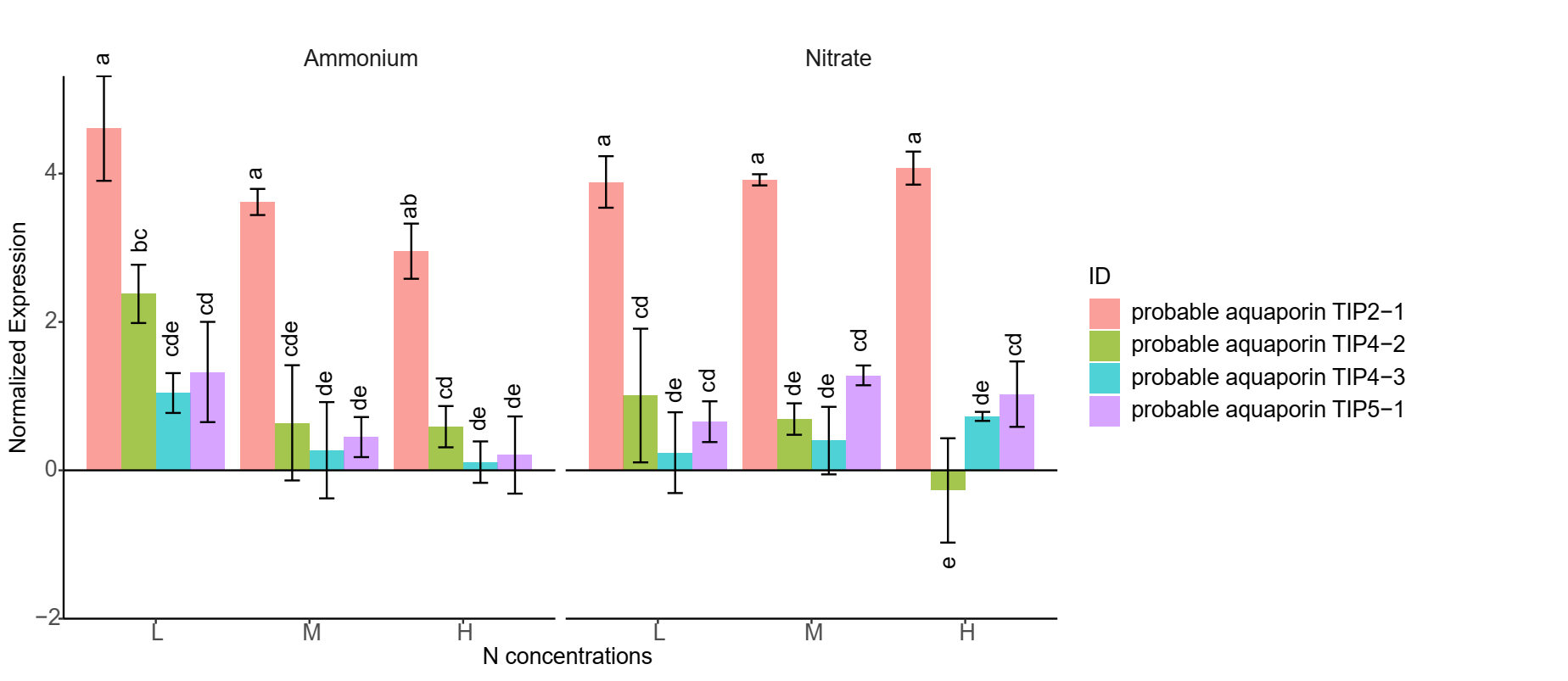
**
